# Alteplase Causing Cardiac Tamponade after Recent Cardiac Pacemaker Placement

**DOI:** 10.5811/cpcem.2018.4.37825

**Published:** 2018-05-18

**Authors:** Corey J. Warf, Martin R. Huecker, Daniel J. O’Brien, Don A. Bertolotti

**Affiliations:** University of Louisville, Department of Emergency Medicine, Louisville, Kentucky

## Abstract

A 56-year-old female presented to the emergency department with evolving cardiac tamponade after receiving alteplase for acute ischemic stroke. This is the first case report of cardiac tamponade from thrombolytics in the setting of recent pacemaker placement. Point-of-care ultrasound was used to make the diagnosis quickly and expedite the patient to the operating room where a pericardial window was performed.

## INTRODUCTION

The risks and benefits of thrombolysis for ischemic stroke are well established, with defined absolute and relative contraindications.^1^ The alteplase package insert lists major surgery (e.g., coronary artery bypass graft, obstetrical delivery, and organ biopsy) as a warning but not an absolute contraindication, while the American Heart Association considers surgery a relative contraindication.^2^ The European Stroke Organization does not list surgery as a contraindication.^3^ Following pacemaker implantation, systemic hemorrhage has occurred following thrombolytic administration,^4^ but there are no reports of pericardial effusion or tamponade.

A history of myocardial infarction (MI) within the prior three months is considered a relative contraindication to the administration of intravenous thrombolytics for acute stroke according to American Heart Association guidelines but not per the European guidelines.^1^,^3^ The risk of thrombolytics to this population includes risk of myocardial hemorrhage predisposing to myocardial wall rupture, pericarditis following MI that may progress to a hemorrhagic pericardial effusion, and embolization from possible ventricular aneurysm.^5^ There are several case reports of hemopericardium and cardiac rupture following thrombolytic therapy for acute stroke.^6^ Only one case had a confirmed prior MI within three months of receiving thrombolytic therapy. None of these cases reported a history of recent cardiac surgery or pacemaker placement.

Pericarditis is not an uncommon complication following pacemaker implantation, with a rate of 2% previously reported in a case series of 395 patients.^7^ Risk of bleeding in the setting of pericarditis is perceived as a potential risk, though this is not supported by the literature.^8^,^9^ There are prior examples of pericardial effusion in the setting of anticoagulation and pacemaker placement.^10^ However, we present a novel case with a combination of factors not previously reported: use of thrombolytics for ischemic stroke; very recent pacemaker placement (less than one week prior); and use of point-of-care ultrasound (POCUS) to quickly make the diagnosis and expedite the disposition of the patient.

## CASE REPORT

We present the case of a 56-year-old female with history of syncope due to third degree atrioventricular heart block presenting initially with onset of stroke symptoms six days after pacemaker placement and two days after hospital discharge. At 5 PM she developed abrupt onset of left facial droop along with left upper and lower extremity weakness. The patient was initially treated at an outlying hospital and received alteplase at 6:35 PM for treatment of acute ischemic stroke.

A chest radiograph performed at the outlying hospital prior to alteplase administration demonstrated an enlarged cardiac silhouette when compared to prior radiographs showing only borderline cardiomegaly. Upon administration, the patient reported mild chest pain and was given nitroglycerine and morphine. Her chest pain resolved and she was transferred to our comprehensive stroke center for admission. The patient presented to our emergency department at 10:10 PM with a heart rate of 122 beats per minute (bpm) and a blood pressure of 109/41 millimeters of mercury (mmHg).

At 11:20 PM the patient went for a computed tomography angiogram (CTA) after an initial assessment by the emergency physician in consultation with the stroke-team attending physician. After CTA at 10:28 PM, she was documented to have a blood pressure of 49/25 mmHg and heart rate of 109 bpm. She was returned to the resuscitation bay for re-evaluation. Cardiac tamponade was suspected due to the extreme hypotension in the setting of thrombolytic administration after recent pacemaker placement.

On reassessment, the patient had become confused with a Glasgow Coma Scale of 14. The emergency physician performed a POCUS, which demonstrated a pericardial effusion with features of cardiac tamponade including diastolic collapse of the right ventricle (Image). At that point the diagnosis of cardiac tamponade was made. The patient was alert and responsive, so an intravenous bolus of normal saline was given while a stat surgical consult was obtained. The surgical team evaluated the patient at the bedside within minutes and was able to review the POCUS findings. As the patient was conscious, they elected to take her immediately to the operating room rather than perform a bedside pericardiocentesis.

While in the operating room, approximately 400 milliliters of coagulated blood were evacuated from the pericardial sac with 150 milliliters of surgical bleeding. The operative report notes resolution of tachycardia following this intervention with heart rate trending down to a range of 80–90 bpm with concomitant improvement in blood pressure. She was discharged two days post-operatively with a pericardial catheter in place. Echocardiogram performed on day of discharge noted a small, residual pericardial effusion.

## DISCUSSION

Although no source of bleeding was identified intra-operatively during the pericardial window, we suspect that a small cardiac perforation occurred during pacemaker placement. Significantly, echocardiogram performed on the day of pacemaker placement immediately following the procedure noted no pericardial effusion. Given the temporal association between the development of tamponade physiology and the administration of alteplase, we believe that administration of thrombolytic medication led to recurrent bleeding and further progression of pericardial hemorrhage and the development of tamponade physiology by the time of the patient’s arrival to our tertiary care center.

**CPC-EM Capsule**

What do we already know about this clinical entity?

Pericardial effusions as a complication of pacemaker placement have been reported in anticoagulated patients but never in the setting of alteplase administration.

What makes this presentation of disease reportable?

We report a novel combination of use of thrombolytics for ischemic stroke, very recent pacemaker placement, and use of bedside ultrasound to diagnose cardiac tamponade.

What is the major learning point?

Cardiac tamponade is a potential risk of administration of alteplase in patients with recent cardiac procedures and should be considered when hypotension is present.

How might this improve emergency medicine practice?

Knowing this complication of alteplase administration may improve early diagnosis of pericardial tamponade in patients with a history of recent cardiac procedures.

It is also noteworthy that point-of-care cardiac ultrasound was performed within 10 minutes of the patient developing hypotension, which led to an immediate management change for this patient. She went immediately to the operating room based entirely on the POCUS findings. One question that remains unresolved is whether pericardial effusion was present prior to alteplase administration. As noted above, the outlying hospital chest radiograph demonstrated an enlarged cardiac silhouette compared to previous imaging at their facility, possibly representing the presence of pericardial effusion. Chest radiograph performed at the facility where pacemaker placement was performed noted stable cardiomegaly, which was followed by two-dimensional echocardiogram where no effusion was seen. Thus, it is unclear whether further change in the cardiac silhouette developed in this six-day span. However, the time between the chest radiograph being performed at the outlying facility and the administration of alteplase was greater than 60 minutes. Had ultrasound been performed at the outlying hospital during this time span, a pericardial effusion may have been identified. This would have been a contraindication to the administration of thrombolytics in this patient.

## CONCLUSION

Though major surgery is listed as a relative contraindication to alteplase, we recommend proceeding with caution in patients who have had any recent surgical procedure. We also stress the importance of assessment with ultrasound in patients at risk for pericardial effusion or cardiac complications from thrombolytic administration. We also recommend considering the use of point-of-care ultrasound to further evaluate patients with abnormal chest radiograph findings if the clinical scenario indicates a possible cardiac etiology. Finally, we highlight the importance of reassessment to recognize complications of high-risk medications such as thrombolytics.

Documented patient informed consent and/or Institutional Review Board approval has been obtained and filed for publication of this case report.

## Figures and Tables

**Image:**
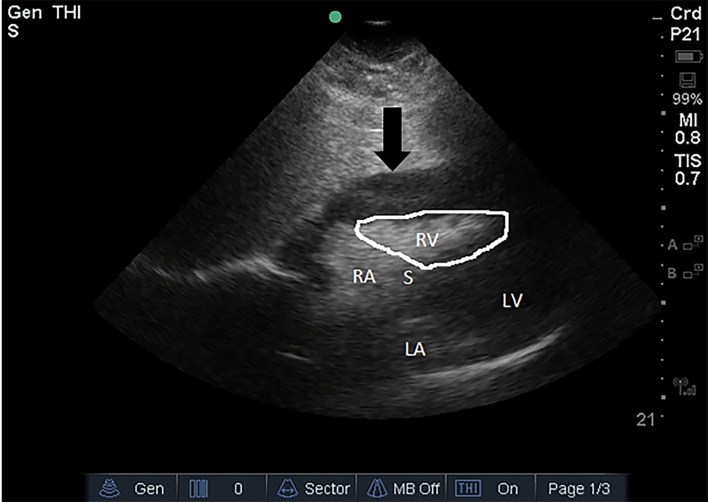
Point-of-care ultrsaound, subxiphoid view of the heart demonstrating a large pericardial effusion (arrow) with diastolic collapse of the right ventricle (outlined). The black arrow points to the pericardium with the large effusion contained within. *RV*, right ventricle; *LV*, left ventricle; *RA*, right atrium; *LA*, left atrium; *S*, intraventricular septum.
